# Diet-Dependent Changes of the DNA Methylome Using a Göttingen Minipig Model for Obesity

**DOI:** 10.3389/fgene.2021.632859

**Published:** 2021-03-11

**Authors:** Y. Feng, S. Cirera, E. Taşöz, Y. Liu, L. H. Olsen, B. Ø. Christoffersen, H. D. Pedersen, T. P. Ludvigsen, R. K. Kirk, C. Schumacher-Petersen, Y. Deng, M. Fredholm, F. Gao

**Affiliations:** ^1^Shenzhen Branch, Guangdong Laboratory for Lingnan Modern Agriculture, Genome Analysis Laboratory of the Ministry of Agriculture, Agricultural Genomics Institute at Shenzhen, Chinese Academy of Agricultural Sciences, Shenzhen, China; ^2^Guangdong Provincial Key Laboratory of Protein Function and Regulation in Agricultural Organisms, College of Life Sciences, South China Agricultural University, Guangzhou, China; ^3^Department of Veterinary and Animal Sciences, Faculty of Health and Medical Sciences, University of Copenhagen, Frederiksberg, Denmark; ^4^Global Drug Discovery, Novo Nordisk A/S, Måløv, Denmark; ^5^Medical Evaluation & Biostatistics, Danish Medicines Agency, Copenhagen, Denmark

**Keywords:** epigenetics, promoter DNA methylation, diet intervention, obesity, metabolism

## Abstract

**Objective:** Environmental factors can influence obesity by epigenetic mechanisms. The aim of this study was to investigate obesity-related epigenetic changes and the potential for reversal of these changes in the liver of Göttingen minipigs subjected to diet interventions.

**Methods:** High-throughput liquid hybridization capture-based bisulfite sequencing (LHC-BS) was used to quantify the methylation status of gene promotor regions in liver tissue in three groups of male castrated Göttingen minipigs: a standard chow group (SD, *N* = 7); a group fed high fat/fructose/cholesterol diet (FFC, *N* = 10) and a group fed high fat/fructose/cholesterol diet during 7 months and reversed to standard diet for 6 months (FFC/SD, *N* = 12). Expression profiling by qPCR of selected metabolically relevant genes was performed in liver tissue from all pigs.

**Results:** The pigs in the FFC diet group became morbidly obese. The FFC/SD diet did not result in a complete reversal of the body weight to the same weight as in the SD group, but it resulted in reversal of all lipid related metabolic parameters. Here we identified widespread differences in the patterning of cytosine methylation of promoters between the different feeding groups. By combining detection of differentially methylated genes with a rank-based hypergeometric overlap algorithm, we identified 160 genes showing differential methylation in corresponding promoter regions in the FFC diet group when comparing with both the SD and FFC/SD groups. As expected, this differential methylation under FFC diet intervention induced de-regulation of several metabolically-related genes involved in lipid/cholesterol metabolism, inflammatory response and fibrosis generation. Moreover, five genes, of which one is a fibrosis-related gene (*MMP9*), were still perturbed after diet reversion.

**Conclusion:** Our findings highlight the potential of exploring diet-epigenome interactions for treatment of obesity.

## Introduction

Obesity is defined as abnormal or excessive fat accumulation that impairs health (WHO). Usually, when an individual is obese it has an increased risk for accompanying comorbidities, such as cardiovascular diseases, dyslipidemia, hypertension, fatty liver disease, type 2 diabetes, and some types of cancer (Bluher, 2019).

Adipose tissue plays a central role in regulation of whole-body energy homeostasis. It is the principal energy reservoir of the body, storing the surplus of dietary energy in the form of lipids in the adipocytes and controlling lipid mobilization to other organs (e.g., liver or muscle). In addition, adipose tissue acts as an endocrine organ releasing adipokines, which communicate with other organs and regulate a range of metabolic pathways (Luo and Liu, 2016). The storage of triglycerides in adipocytes can result in hypertrophy (increase the size of the lipid droplet) and/or hyperplasia (increase in adipocyte number) both resulting in expansion of the adipose tissue and subsequent obesity (Choe et al., 2016). The capacity for storage in the adipocytes is determined by genetic as well as by epigenetic mechanisms (van Vliet-Ostaptchouk et al., 2012). Once the limit for storage is reached, lipids begin to accumulate in other tissues, such as liver and muscle resulting in metabolic disturbances and eventually obesity-related comorbidities. Obesity is associated with an increased risk of non-alcoholic fatty liver disease (NAFLD), characterized by an increased content of triglyceride in the liver (i.e., steatosis) which can also be accompanied by inflammation and fibrosis (i.e., steatohepatitis). However, a proportion of obese individuals do not exhibit metabolic complications such as dyslipidemia or impaired glucose levels and they do not have an increased risk of developing obesity-related comorbidities. This condition has led to the concept of metabolic healthy obese (MHO) (Bluher, 2020). Nevertheless, growing evidence suggests that MHO might be an intermediate stage toward metabolic unhealthy obese (MUO). Yet, the definition and molecular characterization of MHO status is still debated (Schulze, 2019).

Epigenetics has gained attention in the past few years as a hidden layer of the etiology of many disorders, including obesity and cancer (Kasinska et al., 2016). The involvement of epigenetic mechanisms in the development of obesity was first demonstrated in the “Dutch famine birth cohort” study (Lumey et al., 1993). The results obtained from this study, and others that followed, suggest that the intrauterine environment may induce epigenetic marks that may program susceptibility to the development of obesity. Growing evidence suggests that changes in the epigenome induced by the environment and life style (e.g., nutritional habits) may alter gene expression and lead to the development of various diseases, including obesity (Dudley et al., 2011). Moreover, obesity is a phenotypic characteristic of specific imprinted-gene disorders (i.e., Prader-Willi syndrome). One of the main epigenetic mechanisms and the most studied in relation to obesity, is DNA methylation at the promoter region of genes, which plays a pivotal role in the regulation of gene expression (Parrillo et al., 2016). One of the main features of epigenetic modifications is their dynamic nature and reversibility, which provide the potential for development of new therapeutic strategies and advise for changes in lifestyle habits with the aim of preventing and reversing DNA methylation patterns. Indeed, a study in mice submitted to high-fat diet increased methylation levels of *Hoxa5*; however, changing to standard chow let to reversal of the levels of methylation (Parrillo et al., 2016). Conversely, it seems that not all epigenetic marks can be reversed to basal levels. Indeed, another study using high fat diet to induce obesity in mice showed that despite normalization of most metabolic parameters and body weight after diet switch, hepatic inflammation and steatosis persisted (Fischer et al., 2018). Similar results were also observed for several key liver genes, such as the lipid-regulating gene *Apoa4*, which remained hypomethylated even after introduction to normal chow diet (Kim et al., 2019). Thus, it appears that obesity contributes with a metabolic fingerprint that persists in liver and adipose tissue through weight loss and predisposes to the development of the metabolic syndrome upon weight regain (Fischer et al., 2018).

Pigs are genetically and physiologically close to humans (Schook et al., 2015). Therefore, several breeds of pigs, including Ossabaw and Göttingen Minipigs (GM) have been extensively used as animal models for MS and obesity. Specifically, we have worked with the GM breed as an obesity model, providing evidence of a genetic predisposition for an MHO-like phenotype in this breed (Frederiksen et al., 2017). We have further shown that GM fed a high fat diet become obese but do not develop hepatic steatosis (Schumacher-Petersen et al., 2019), and that severely obese GM have a large capacity for adipose tissue expansion and are protected against many of the metabolic and hepatic abnormalities associated with obesity (Cirera et al., 2020). In the present study, we have compared the promoter methylome in liver tissue of GM fed a high fat/fructose/cholesterol (FFC) diet with the promotor methylome of GM fed the FFC diet and reverted to standard chow (FFC/SD), and GM fed standard chow (SD), respectively in order to see a possible influence of diet in gene regulation mechanisms. We showed that there is extensive perturbation of the methylome of promoter regions in genes involved in metabolism and immune response pathways in the GM subjected to the FFC diet compared to the SD and the FFC/SD group of pigs. Surprisingly, the diet-induced methylome variation could only partially be reversed to the lean stage after replacing the FFC diet to the SD diet. Indeed, promotors of several obesity-related genes, such as fibrosis-related gene *MMP9*, remained differentially methylated and their expression was still perturbed after 6 months of diet reversion.

## Materials and Methods

### Minipig Model

Male GM (Ellegaard Göttingen Minipigs A/S, Dalmose, Denmark) (*N* = 54 in total) aged 6–7 months were castrated (in order to avoid undesired sexual behavior in sexually mature boars), weight stratified and distributed into four treatment groups. Animals were housed at the Experimental Animal Unit at University of Copenhagen and fed individually once daily for 13 months [described in Andreasen et al. (2018) and Schumacher-Petersen et al. (2019)]. For the present study, 29 pigs assigned into three groups were selected. The included groups were: lean control pigs (SD, *N* = 7) fed standard minipig chow (Minipig, SDS, UK); a group fed high fat/fructose/cholesterol (2%) diet (5B4L) for the first 5 months and subsequently changed to a similar diet with 1% cholesterol (9G4U) for the remainder of the 13 months study period (TestDiets®, Missouri, USA) (FFC, *N* = 10); a diet normalization group fed fat/fructose/cholesterol for the first 7 months and then returned to standard minipig chow fed 500 g/day for the remaining 6 months of the study (FFC/SD, *N* = 12). A detailed feeding protocol can be seen in Figure 1 in Andreasen et al. (2018). Pathological hepatic changes of these minipigs were characterized in a paralleled study (Schumacher-Petersen et al., 2019) and gene expression profiling of the SD and FFC groups is included in Cirera et al. (2020). Basic morphometric and metabolic characteristics of the selected minipigs are described in Table 1.

**Table 1:** Morphometric and metabolic features of the minipigs.

**Diet group**	**SD (*N* = 7)**	**FFC/SD (*N* = 12)**	**FFC (*N* = 10)**	***P*-value**
BW (kg)	39.5 (38.2; 40.7)	55.0 (52.5; 59.0)^a^	79 (77; 81)^b^^,^^c^	1.79e-15
TBF (%)	27.65 (27.30; 29.95)	48.10 (44.25; 50.25)^a^	64.8 (63.4; 67.6)^b^^,^^c^	5.26e-12
TC (mmol/L)	1.70 (1.65; 2.06)	1.87 (1.56; 2.0)	12.37 (11.18; 15.56)^b^^,^^c^	2.64e-11
TG (mmol/L)	0.34 (0.30; 0.35)	0.39 (0.33; 0.45)	0.82 (0.63; 1.17)^b^^,^^c^	1.92e-4
GLU (mmol/L)	3.48 (3.32; 3.66)	3.72 (3.47; 3.88)	3.75 (3.60; 3.90)	0.304
LW (kg)	0.51 (0.46; 0.56)	0.73 (0.66; 0.77)^a^	2.15 (1.09; 2.27)^b^^,^^c^	7.38e-07
GLC (mg/g)	15.30 (3.96; 31.44)	11.77 (6.62; 22.79)	20.19 (15.74; 29.12)	0.546
LTG (mg/g)	12.23 (9.03; 13.96)	14.14 (12.45; 17.87)	13.70 (11.66; 16.86)	0.977
CHOL (mg/g)	3.51 (3.34; 3.56)	3.58 (3.45; 3.90)	18.23 (11.95; 23.94)^b^^,^^c^	3.29e-07

*Results are presented as median and 25 and 75% quartiles*.

*BW, body weight; TBF, total body fat; TC, total cholesterol; TG, triglycerides; GLU, plasma glucose; LW, liver weight; GLC, glycogen content in liver; LTG, triglycerides content in liver; CHOL, cholesterol content in liver*.

a *Significantly different from SD*.

b *Significantly different from SD*.

c *Significantly different from FFC/SD*.

*The one-way ANOVA test was performed using “aov” package in R (version 3.6.2) for the three diet feeding groups. Parameters with Benjamini-Hochberg corrected P-value < 0.05 were considered significant*.

*These baseline, metabolic, and morphometric parameters have been described previously (Andreasen et al., 2018; Schumacher-Petersen et al., 2019; Cirera et al., 2020)*.

All the minipigs had free access to bedding material and fresh drinking water at all times. Before termination, the minipigs were fasted overnight and then euthanized by exsanguination in general anesthesia by a mixture of zolazepam, tiletamine, ketamine, xylazine, and butorphanol as described by a previous study (Pedersen et al., 2014). Samples from left medial liver lobe were collected, snap frozen in liquid nitrogen and kept at −80°C for subsequent analysis of the DNA methylation and expression studies.

The animals included in this study were a part of a larger study (Andreasen et al., 2018; Schumacher-Petersen et al., 2019). The study was approved by the Animal Experiment Inspectorate, Ministry of Justice, DK.

### Promoter-Targeted LHC-BS Analysis

Genomic DNA was extracted from the liver tissue using the DNeasy Blood and Tissue Kit (Qiagen, Germany) according to the manufacturer's instructions. The purified genomic DNA was subjected to promoter-targeted bisulfite conversion library construction based on previous protocols, with minor changes (Gao et al., 2014). Regions from 2.2 kb upstream to 0.5 kb downstream of the transcriptional start sites were denoted as promoters. Briefly, 1 μg of the isolated genomic DNA was fragmented to a mean size of 200–300 bp by the Covaries S2 system. After purification, the products were further treated to repair blunt end by a mix of T4 DNA polymerase, Klenow Fragment and T4 polynucleotide kinase. After addition of dA to the 3′ end and index sequence ligation, the reaction products were purified using QIAquick PCR purification kit (Qiagen, Germany). 250 ng DNA from each of three adapter-ligated libraries were equally pooled together for subsequent liquid hybridization capture procedure as previously described (Gao et al., 2014). After addition of unmethylated lambda DNA into each library, the captured products were bisulfite converted using EZ DNA Methylation-Gold Kit^TM^ (ZYMO Research, CA, USA) following the manufacturer's instructions. PCR products were purified using AMPure beads (Agilent, USA) and quantified by the Bioanalyzer analysis system (Agilent, USA) and real-time PCR assay. The final libraries were subjected to pair-end 150 bp sequencing using Illumina Hiseq 2500 platform.

After base calling, sequencing reads with more than 30% “N”s or quality value <20 in over 10% of the sequences were discarded. The clean reads were aligned to the pig reference genome (NCBI build Sscrofa11.1) by the bisulfite sequence MAPping program (BSMAP) (Xi and Li, 2009) with up to 4 mismatches and 2 continuous gaps. The methylation level of CpG sites was calculated as the ratio of the methylation-supportive read number to the total read number of individual cytosines. Average methylation level of genes was computed as the sequenced depth of methylated CpGs divided by total sequenced depth of CpGs in corresponding genomic region. To ensure the accuracy of measurement, a minimal 4× read number was used for further analysis.

### Identification of DMRs/DMGs

Putative differentially methylated regions (DMRs) and genes (DMGs) were identified as follows: Firstly, metilene software (v0.2-7) (Juhling et al., 2016) was used to identify DMRs. We used the following parameters: distance between two adjacent CpGs ≤300 bp, ≥5 CpGs, and with an absolute methylation difference >0.1 and passing through Bonferroni correction (*P* < 0.05). Subsequently, genes that contained at least one DMR, of which >50% of sequences were overlapped with the putative promoters and exhibited differential methylation in corresponding promoter regions (Mann-Whitney *U*-test, *P* < 0.05) were identified as differentially methylated genes (DMGs).

### Overlap of Methylation Profiles

We used the Rank-Rank hypergeometric overlap (RRHO) test to compare the trend of methylation alteration between different diet groups. The RRHO algorithm is a threshold-free approach that can detect overlap trends between particular subsets of genes by hypergeometric distribution based on several metrics, such as a signed log_10_-transformed *P*-value and fold-change (Cahill et al., 2018). In this study, the methylation profiles from two data sets were compared by a signed log_10_-transformed Mann-Whitney *U*-test *P*-value based on the mean methylation level of corresponding regions. By these metrics, genes with significantly increasing methylation level will be placed at the top of a ranked list while genes with significantly decreasing methylation level will be placed at the bottom with all relatively unchanging genes in the middle (Supplementary Figure 1). To confirm the results of RRHO, we further used Spearman's Rank-Order Correlation to measure the strength and direction of methylation trend based on the effect size of overlap regions identified by RRHO test.

### qPCR Validation

High-throughput quantitative real-time PCR (qPCR) experiment was conducted on the Biomark HD system as previously described (Cirera et al., 2020). Briefly, RNA was isolated from liver tissue using the Tri® Reagent protocol (MRC Gene, Cincinnati, OH 45212 USA) with DNAse treatment included (Qiagen, Germany). A mixture of 0.5 μl Improm-II^TM^ reverse transcriptase (Promega, Madison, USA), 0.25 μg 1:3 OligodT/Random hexamer primers, 2 μl 5× ImProm-II buffer, 10 units Rnasin® Ribonuclease inhibitor (Promega, Madison, USA), 2.5 mM MgCl_2_ and 2 mM dNTP was used for cDNA synthesis. “Pick Primers” tool embedded in PubMed was used to design primer sequences for relevant liver genes including most of the splice variants. The diluted cDNA was first pre-amplification using TaqMan PreAmp Master Mix (Life Technologies, Nærum, Denmark) for 15 cycles and then cleanup with Exonuclease I (New England BioLabs, Herlev, Denmark) following the Fluidigm's protocol (Fluidigm PN 100-5875 C1) with minor modifications. The primer concentrations in the primer pool were 250 nM. *TBP* and *ACTB* were chosen as reference genes for data normalization based on Cirera et al. (2020). GenEx 6.0 software (MultiD, Sweden) was used to pre-process the raw qPCR data, including PCR efficiency correction, normalization and conversion to relative quantities. The genes profiled by qPCR in this project are a subset of the genes profiled in Cirera et al. (2020) and the primer sequences are listed in Table 2.

**Table 2:** Subset of primer sequences for qPCR validation.

**Gene**	**Forward primer**	**Reverse primer**
ACTB	TCTGGCACCACACCTTCT	TGATCTGGGTCATCTTCTCAC
TBP	AACAGTTCAGTAGTTATGAGCCAGA	AGATGTTCTCAAACGCTTCG
APOA4	AGATGTTCTCAAACGCTTCG	TAGCCACTTGGTCGGCATTG
APOB	TGTCCAAGTACGAGCTCAGG	GCAGGAGGGCAGAAATGATG
COL1A1	CAACGAGATCGAGATCCGGG	TTCGATCACTGTCTTGCCCC
HMGCR	GACTCCGTTGACTGGAGACG	AAAGAGGCCATGCATTCGGA
KLB	TGGTTCACAGACAGTCACGT	TGCCATTCAAAGCCATCCAG
LDLR	GCAGTGCGACAGGGAATATG	ACACTTGAACTTGTTGGGCC
MMP9	CGGGAGACCTACGAACCAAT	TCCAGGGACTGCTTTCTGTC
MTTP	TCCCATTGCTCCTGAAGTACG	ACGCACAGTCTTTTCGTGAAC
TLR4	AGAACTGCAGGTGCTGGATT	TGGATAGGATTTCCCGTCAG
TNF	CCCCCAGAAGGAAGAGTTTC	CGGGCTTATCTGAGGTTTGA

### Statistical Analysis

Principal component analysis (PCA) was performed using “ggbiplot” package in R (version 3.6.2) based on the mean methylation level of promoter regions of the three diet groups. Methylation levels of individual promoter regions among different diet groups were compared using the Mann-Whitney *U*-test. DNA methylation fold-change (FC) was calculated as ratio of mean methylation levels of the two diet groups under consideration. Promoters with a multiple-testing corrected *P* < 0.05 were considered as statistically significant. Subsequently, the Database for Annotation, Visualization and Integrated Discovery (DAVID, v6.8) was used to determine potential gene ontology (GO) and enrichment analysis of differentially methylated genes (Huang da et al., 2009). Terms with Benjamini-Hochberg corrected *P* < 0.05 were defined as significantly enriched terms and pathways. The chi-square analysis was performed using ‘chisq.test’ package in R (version 3.6.2) based on the mean methylation level of promoter regions of three diet groups. Expression levels of genes between different diet groups were compared using *t*-test in R (version 3.6.2), and FC >1.5 or < -1.5 and adjusted-*P*-values <0.05 were reported as differentially expressed genes.

## Results

### Morphometric and Metabolic Changes in GM Due to Diet Intervention

As seen in Table 1, the FFC diet had a high impact on both morphometric and metabolic phenotypes in the GM. The pigs in the FFC diet group became morbidly obese as documented by the highly significant increase in body weight (BW) and total body fat (TBF). All the measured metabolic traits increased significantly except for liver content of glycogen (GLC) and triglycerides (LTG) and plasma glucose (GLU). The FFC/SD diet did not result in a complete reversal of the body weight/composition to the same weight or TBF as in the SD group, but it resulted in reversal of all lipid related metabolic parameters. Some of the measurements have been included in previous studies (Andreasen et al., 2018; Schumacher-Petersen et al., 2019).

### Methylome Comparison Between GM Subjected to Diet Intervention

**Figure 1:**
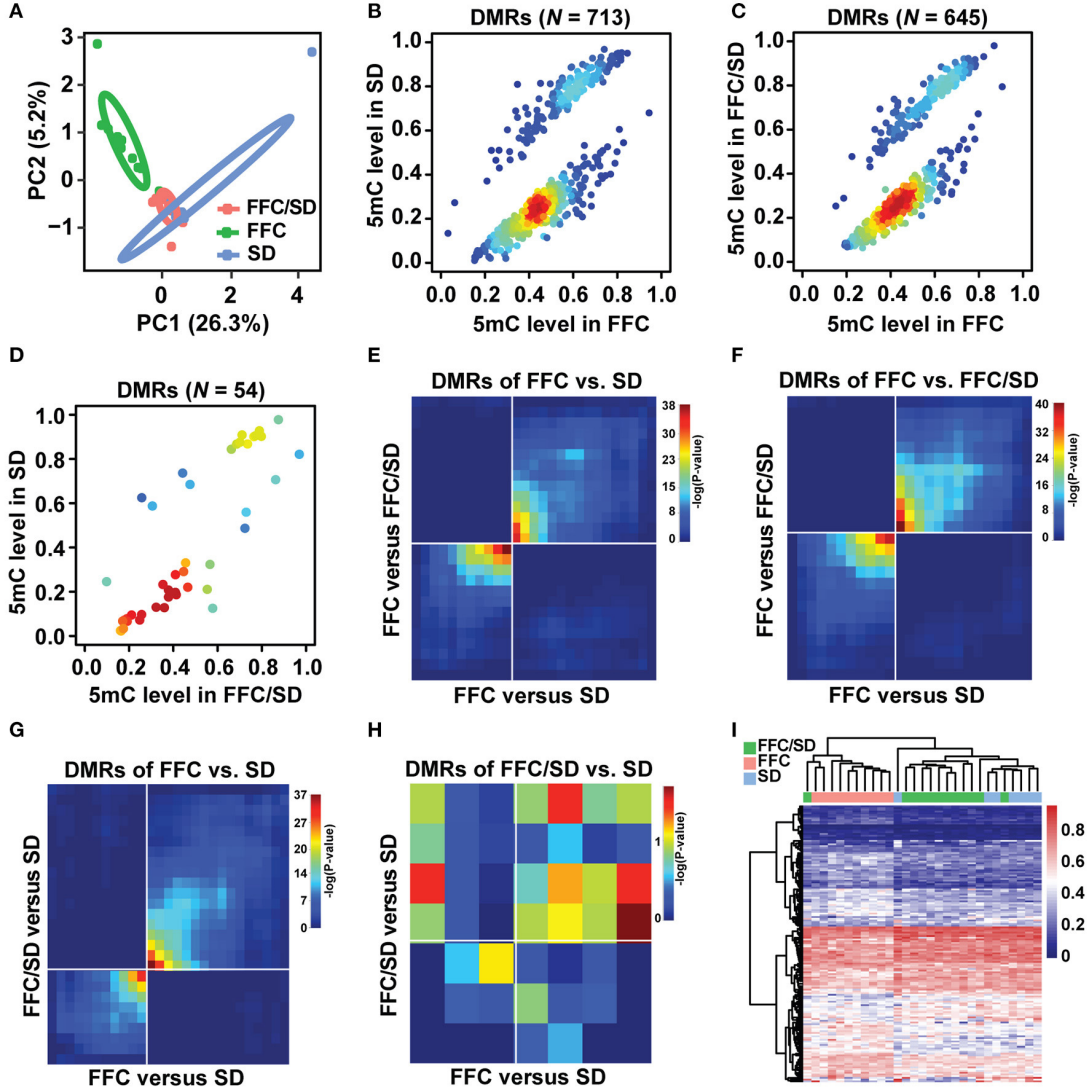
Methylome comparison between different diet fed pigs. **(A)** Principal component analysis based on 408,458 methylated common CpG sites of three diet groups. Circles representing the three diet feeding groups could be separated at a confidence interval of 68%. **(B–D)** Scatter plot of methylation level of DMRs in FFC vs. SD **(B)**, FFC vs. FFC/SD **(C)** and FFC/SD vs. SD **(D)** diet fed pigs, with density at each point (red for high density, blue for low density). **(E–H)** RRHO maps comparing methylation profiles between FFC vs. SD and FFC vs. FFC/SD across DMRs of FFC vs. SD **(E)** and in FFC vs. FFC/SD **(F)**, and between FFC vs. SD and FFC/SD vs. SD across DMRs of FFC vs. SD **(G)** and of FFC/SD vs. SD **(H)**. **(I)** Heatmap depicting the hierarchical clustering of methylation level of promoter regions of core genes (*N* = 160) that exhibited significant overlap between FFC vs. SD and FFC vs. FFC/SD.

The status of cytosine methylation in the promoter regions in the three diet groups was examined by LHC-BS. A total of 32,163 probes were captured based on the pig reference genome (NCBI build Sscrofa11.1), representing 4.20 million CpG dinucleotides in the promoters and/or a coverage of 29,403 (97.0%) genes in the Ref-Seq database. We obtained an average of 8.9 Gb raw data for each sample, among which 81.7% could be mapped uniquely to the pig reference genome after quality control. Accordingly, 51.73% of these uniquely aligned reads were located at defined promoter regions. Approximately 28,680 (~94.65%) promoters were covered by at least 1× read depth (Supplementary Table 1). To accurately characterize the methylation level, a minimum of 4× sequencing depth was used in each sample, enabling an average of 2.34 mio CpG coverage per sample and a total of 408,458 common methylated CpG sites for the three diet groups. For each diet group, we conducted a Pearson's correlation analysis based on the 408,458 common CpG sites and observed a high concordance of cytosine methylation status between individuals (*r* > 0.85; Supplementary Figure 2). Averagely, the mean methylation levels were 60.13, 58.78, and 59.29% in SD, FFC/SD and FFC diet fed pigs (Supplementary Table 1). Then, we performed principal component analysis based on the 408,458 common CpG sites in the promoter region. As seen in Figure 1A, the three diet groups were classified into two clusters, that is, the SD and FFC/SD diet groups clustered together and separately from the FFC diet group. Similar results were also obtained via hierarchical clustering analysis (Supplementary Figure 3), which was performed on the top 1,000 genes containing highly variable promoter methylations based on the *P*-values obtained from chi-square analysis.

The flow chart in Supplementary Figure 4 describes the strategy we used to analyze the methylome changes between the different diet groups. We first identified DMRs in the following comparisons: FFC vs. SD, FFC vs. FFC/SD, and FFC/SD vs. SD diet groups as the principal component analysis suggested. In each comparison, CpGs with more than 30% missing values in each diet group were removed from subsequent analysis. As a result, 713 DMRs were identified in the FFC vs. SD diet groups, among which 497 DMRs exhibited hypermethylation and 216 exhibited hypomethylation (Figure 1B). Likewise, 645 DMRs were identified in the FFC vs. FFC/SD diet groups, among which 453 DMRs exhibited hypermethylation and 192 exhibited hypomethylation (Figure 1C). In contrast, only 54 DMRs were identified in the FFC/SD vs. SD diet groups, among which 35 DMRs exhibited hypermethylation and 19 exhibited hypomethylation (Figure 1D). To identify DMRs that changed with uniform direction due to the FFC diet, we made two comparisons: FFC vs. SD and FFC vs. FFC/SD, using the RRHO test approach. The methylation profiles of FFC vs. SD and FFC vs. FFC/SD were compared and ranked against (i), the list of DMRs identified between FFC and SD (713), and against (ii), the list of DMRs identified between FFC and FFC/SD (645). We identified 494 and 457 DMRs changed in the same direction in (i) and (ii), respectively (see Figures 1E,F), i.e., in total 951 DMRs. We also applied the RRHO test to identify loci that remained differentially methylated even after diet reversion. This was done by comparing the methylation profiles of FFC vs. SD and FFC/SD vs. SD and ranking against (iii) the list of DMRs identified between FFC and SD (713) and against (iv) the list of DMRs identified between FFC/SD and SD (54). We identified 152 and 13 DMRs ranked in the same direction in (iii) and (iv), respectively (see Figures 1G,H), i.e., a total of 165 DMRs. We further confirmed these results using Spearman's correlation analysis based on effect size in corresponding overlapped DMRs. Consistent with the coordinated overlap in DMRs reported by RRHO, there was a strong positive Pearson's correlation between the FFC/SD and the SD pigs in the overlapping DMRs of FFC vs. SD diet fed pigs (*r* = 0.31; *P*-value < 2.2e-16) and in the overlapping DMRs of the FFC vs. FFC/SD pigs (*r* = 0.33; *P*-value < 2.2e-16). Concordant results were observed in the FFC and the FFC/SD pigs for the overlapping DMRs of the FFC vs. SD diet fed pigs (*r* = 0.33; *P*-value < 2.2e-16) and in the overlapping DMRs of the FFC/SD vs. SD pigs (*r* = 0.32; *P*-value < 2.2e-16).

Subsequently, we characterized the DMGs in the 951 DMRs that changed in the same direction due to the FFC diet. Herein, we observed that 655 non-redundant genes containing one or more than one DMR in the putative promoter regions. Among the 655 genes identified, we identified a panel of 160 or 99 genes that showed differential methylation in corresponding promoter regions either in the SD or in the FFC/SD group when comparing with the FFC group (*P* < 0.05). Thus, we merged the two DMG datasets and obtained a final panel of 160 DMGs for subsequent correlation and pathway analysis (Figure 1I, |FC| = 1.06–2.60, Supplementary Table 2). To investigate whether the methylation levels of these 160 genes showed associations with known morphometric and metabolic parameters, we conducted Pearson's correlation analysis. Specifically, we observed that most of these genes showed significant correlation of methylation level with five morphometric and metabolic features, including body weight (BW), total body fat (TBF), total cholesterol (TC), liver weight (LW), and cholesterol content in liver (CHOL) (*P*-value ≤ 0.01 **|** |*r*| ≥ 0.4); of these, BW and TBF showed the highest correlation (*P* value ≤ 0.001 **|** *r* ≥ 0.6) (Supplementary Table 3). Then we assessed whether epigenetic changes due to dietary intervention could be related to specific obesity-related metabolic pathways using DAVID. A total of 58 DMGs out of 160 were categorized into 39 major gene ontology terms (Supplementary Table 4). The biological process (BP) of these DMGs mainly embodied “positive regulation of humoral immune response mediated by circulating immunoglobulin” (*P* = 2.7E-4), “cholesterol biosynthetic process” (*P* = 6.05E-4), “inflammatory response” (*P* = 0.01), “sterol biosynthetic process” (*P* = 0.039), and “phospholipid transport” (*P* = 0.065). To further explore the functional significance of these DMGs, we also conducted KEGG pathway analysis. Using a cutoff of adjusted-*P-*value ≤ 0.05, we revealed 10 significantly enriched pathways (Supplementary Table 3). The top five functional annotations were “PI3K-Akt signaling pathway” (*P* = 5.7E-4), “NF-kappa B signaling pathway” (*P* = 4.0E-3), “Transcriptional mis-regulation in cancer” (*P* = 5.2E-3), “TNF signaling pathway” (*P* = 8.2E-3), and “Insulin resistance” (*P* = 9.3E-3). Many of these processes and pathways bear relevance to the biology and etiology underlying obesity initiation and progression.

Among the panel of 160 DMGs identified above, we observed that methylation levels of the majority (*N* = 125, *P* > 0.05) in the FFC/SD group were comparable to that identified in the SD group; however, certain genes (*N* = 35) still maintain altered methylation levels even after reversing the diet to normal chow (Supplementary Table 2), suggesting that the FFC diet-induced obesity phenotype was only partially reversed by normal chow. This result encouraged us to detect genes that were differentially methylated in the FFC or in the FFC/SD diet groups in comparison to SD diet group (*P* < 0.05), whereas methylation levels between FFC and FFC/SD diet groups were equivalent, by using the 165 DMRs found in the RRHO test. As a result, we observed 151 non-redundant genes that contained one or more than one DMR in the putative promoter regions. Further, among these genes we identified five genes, including a long non-coding RNA *LOC110255742* (FC = −1.66 in FFC vs. SD diet and FC = −1.03 in FFC/SD vs. SD diet), Matrix metallopeptidase 9 (*MMP9*, FC = −1.12 in FFC vs. SD diet and FC = −1.01 in FFC/SD vs. SD diet), Serpin Family B Member 1 (*SERPINB1*, FC = −1.16 in FFC vs. SD diet and FC = −1.05 in FFC/SD vs. SD diet), Synaptotagmin Like 3 (*SYTL3*, FC = −1.14 in FFC vs. SD diet and FC = −1.05 in FFC/SD vs. SD diet) and VAC14 Component of PIKFYVE Complex (*VAC14*, FC = −1.54 in FFC vs. SD diet and FC = −1.01 in FFC/SD vs. SD diet) (Figures 5A–E), that exhibited such epigenetic features in corresponding promoter regions.

Taken together, we identified a panel of genes that showed differential methylation in the FFC diet group (comparison between FFC vs. SD and FFC vs. FFC/SD) and after diet reversion (comparison between FFC vs. SD and FFC/SD vs. SD). To provide evidence for the functional impact of the diet-induced changes of the methylome we have characterize selected genes and pathways of potential importance for the morphometric and metabolic changes caused by the diet intervention.

### Genes Involved in Lipid/Cholesterol Metabolism

**Figure 2:**
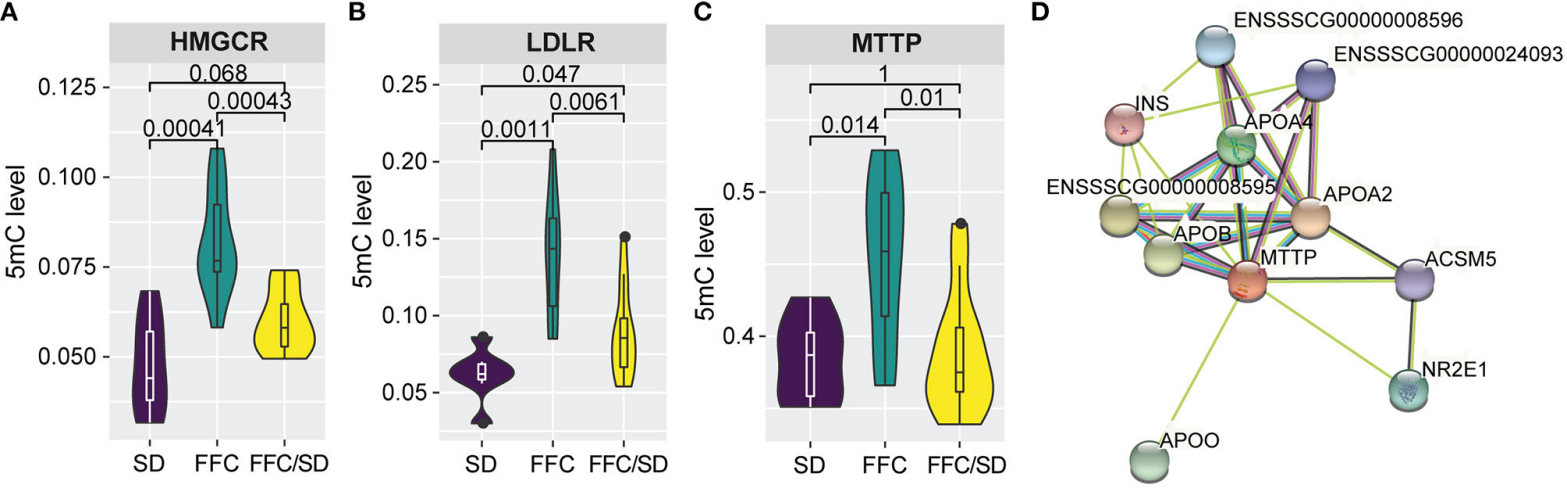
Methylation changes associated with lipid/cholesterol metabolism. **(A–C)** Boxplot of methylation level of promoter regions of *HMGCR* **(A)**, *LDLR* **(B)**, and *MTTP* **(C)**. *P*-values were calculated using Mann-Whitney *U*-test. **(D)** Construction of protein-protein interaction networks using STRING database (v11.0). Edges represent protein-protein association.

In this study, five genes involved in cholesterol biosynthetic process were identified to be differentially methylated in both the SD and the FFC/SD group when comparing with the FFC group, including *APOA1, HMGCR, INSIG1, PRKAA2, LDLR* (Supplementary Table 3). Notably, we observed that HMC CoA reductase (HMGCR), the rate-limiting enzyme in cholesterol synthesis, was significantly hypermethylated in the FFC group (Figure 2A, FC = 1.72 in FFC vs. SD diet and FC = 1.36 in FFC vs. FFC/SD diet). A similar trend was observed in promoter regions of low-density lipoprotein receptor (LDLR, FC = 2.25 in FFC vs. SD diet and FC = 1.56 in FFC vs. FFC/SD diet), which is another major point of control in cholesterol homeostasis (Figure 2B). As promoter hypermethylation is often associated with heterochromatin state and transcriptional repression, we investigated the mRNA expression level of these genes by qPCR. Significantly decreased expression was observed in the FFC group when compared to SD and FFC/SD groups in *HMGCR* (FC = −12.41 in FFC vs. SD diet and FC = −10.61 in FFC vs. FFC/SD diet; *P* < 0.001) (Table 3) and *LDLR* (FC = −5.49 in FFC vs. SD diet and FC = −6.43 in FFC vs. FFC/SD diet; *P* < 0.001) (Table 3), which is consistent with the metabolic changes showing that significantly higher cholesterol level was observed in the FFC diet group (Table 1) (Schumacher-Petersen et al., 2019). Together the results suggest that epigenetic mechanisms play a role for the dysregulation of cholesterol metabolism observed under high fat/fructose/cholesterol intake.

**Table 3:** QPCR results on DMGs.

**Genes**	**FFC vs. SD**		**FFC vs. FFC/SD**		**FFC/SD vs. SD**	
	**FC**	***P*-value**	**FC**	***P*-value**	**FC**	***P*-value**
**Genes involved in lipid/cholesterol metabolism**						
**HMGCR**	−12.41	3.17*E*−05	−10.61	1.05*E*−06	−0.81	0.293
**LDLR**	−5.49	4.33*E*−05	−6.43	2.44*E*−07	1.15	0.289
**MTTP**	−1.61	1.72*E*−03	−1.51	2.89*E*−04	−0.91	0.470
APOB	−1.46	3.95*E*−03	−1.28	8.35*E*−03	−0.67	0.432
APOA4	1.28	0.035	−1.01	0.781	1.26	0.032
**Genes involved in pro-inflammatory response**						
TNF	−0.82	0.155	1.03	0.205	−0.64	0.241
**TLR4**	2.03	3.90*E*−04	2.01	1.10*E*−05	1.02	0.732
**Genes involved in fibrosis generation**						
**COL1A1**	2.68	0.021	1.92	0.03	1.21	0.587
**KLB**	−20.68	9.44*E*−06	−13.87	3.54*E*−06	−0.83	0.756
**Genes still perturbed after diet reversion**						
**MMP9**	6.48	1.13*E*−03	3.75	2.7*E*−03	3.71	8.3*E*−03

*FC, fold-change; Relative expression used here was log2 transformed and presented as mean values ± standard error of the mean; Genes highlighted in bold have FC >1.5 or FC < -1.5 and Benjamini-Hochberg adjusted P <0.05; A positive FC represents that the gene had increased expression levels in the first group of the respective comparison, and a negative FC value represents that the gene had decreased expression levels in the first group of the respective comparison*.

Furthermore, of particular interest is the differentially methylated genes involved in phospholipid transport. Apart from the aforementioned *LDLR*, the microsomal triglyceride transfer protein (MTTP) is also included in this biological process. Here, we observed that in the FFC diet group, *MTTP* showed significantly higher methylation level in promoter regions than that detected in the SD or FFC/SD groups (Figure 2C, FC = 1.18 in FFC vs. SD diet and FC = 1.17 in FFC vs. FFC/SD diet). To further identify potential interactors of *MTTP*, we searched the STRING database to infer functional protein-protein interaction networks. The results showed that MTTP have been reported to interact with several APO gene family members including apolipoprotein B (APOB) and apolipoprotein A4 (APOA4) (Figure 2D). These genes have been demonstrated to be lipid biomarkers mediating the MHO phenotype (Doumatey et al., 2016). Therefore, we also examined the expression level of these genes. We found decreased mRNA expression levels of *MTTP* (FC = −1.61 in FFC vs. SD diet and FC = −1.51 in FFC vs. FFC/SD diet; *P* < 0.01) (Table 3) and *APOB* (FC = −1.56 in FFC vs. SD diet and FC = −1.38 in FFC vs. FFC/SD diet; *P* < 0.01) (Table 3) in the FFC diet group compared to the other groups, whereas no obvious change was detected in expression of *APOA4* (Table 3). Collectively, these results suggest that the FFC diet challenged the lipid/cholesterol metabolism in the liver.

### Genes Involved in Pro-inflammatory Response

**Figure 3:**
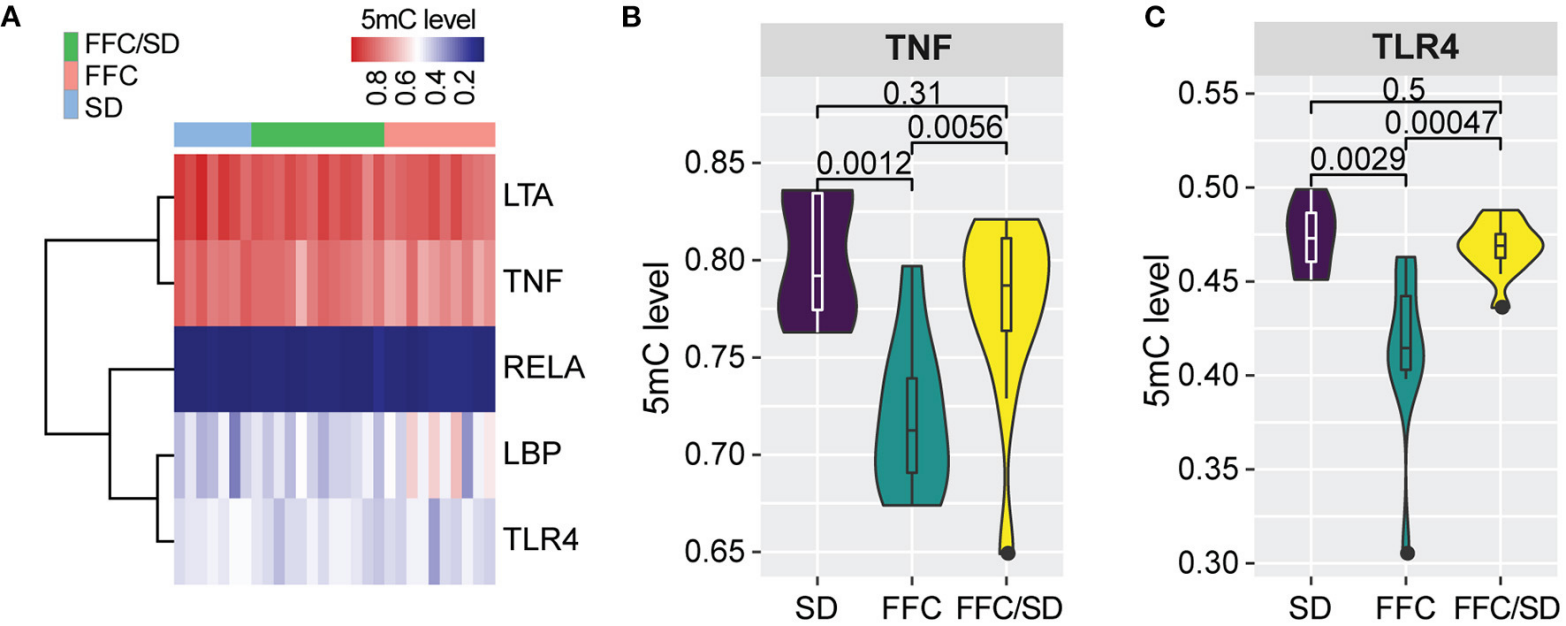
Methylation changes associated with inflammation response. **(A)** Heatmap depicting the hierarchical clustering of methylation level of promoter regions of the five genes associated with inflammation response. **(B,C)** Boxplot of methylation level of promoter regions of *TNF* **(B)** and *TLR4* **(C)**. *P*-values were calculated using Mann-Whitney *U-*test.

Based on KEGG analysis, we observed that genes associated with “NF-kappa B signaling pathway” and “Toll-like receptor signaling pathway” which are pro-inflammatory pathways, were differentially methylated among the three diet groups. Five genes involved in this pathway were investigated: lipopolysaccharide binding protein (*LBP*), lipoteichoic acid (*LTA*), nuclear factor NF-kappa-B p65 subunit (*RELA*), tumor necrosis factor alpha (*TNF*), and toll-like receptor 4 (*TLR4*) (Figure 3A). Compared with the SD diet group, the methylation level in the promoter region of *TNF* was decreased by 8.7% in the FFC/SD group and 10.3% in the FFC group (Figure 3B). The methylation level for the promoter region for *TLR4* in the FFC diet group was decreased by 14.7 and 13.3% in comparison to the SD and FFC/SD groups, respectively (Figure 3C). We then used qPCR to validate the impact of differential methylation on two of the above-mentioned genes. With respect to *TNF*, no significant change in mRNA expression level was detected in the FFC diet group when comparing with the SD and FFC/SD groups (Table 3). However, the expression level of *TLR4* in the FFC/SD group was comparable to that detected in the SD group, whereas both were significantly lower than that in the FFC group (FC = 2.03 in FFC vs. SD diet and FC = 2.01 in FFC vs. FFC/SD diet; *P* < 0.001) (Table 3).

### Genes Involved in Fibrosis Generation

**Figure 4:**
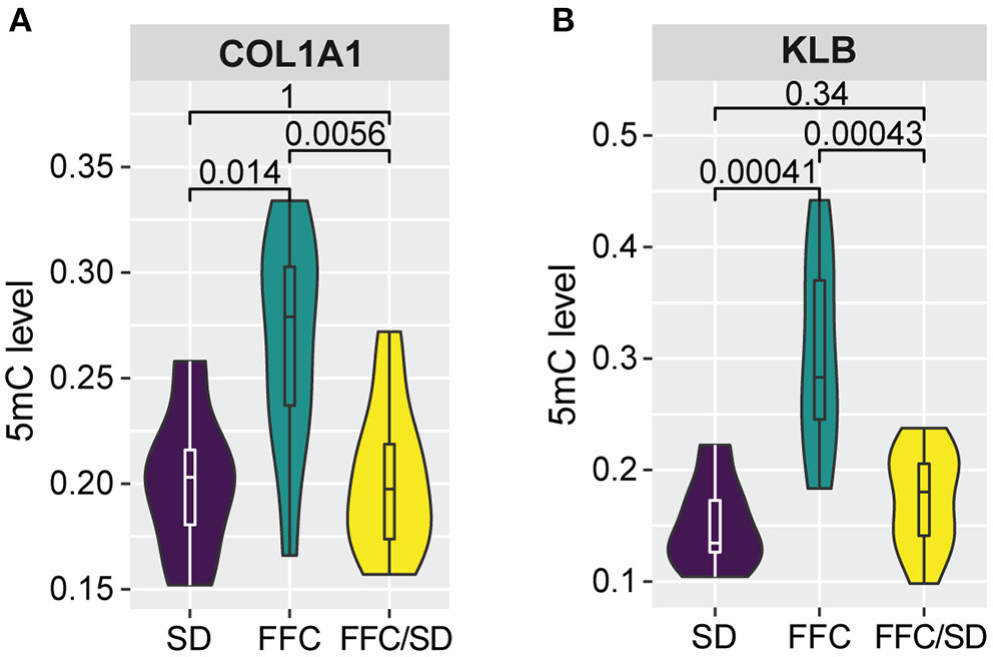
Methylation alterations associated with fibrosis generation. **(A,B)** Boxplot of methylation level of promoter regions of *COL1A1* **(A)** and *KLB* **(B)**. *P*-values were calculated using Mann-Whitney *U*-test.

The increased inflammation and impaired lipid and cholesterol metabolism may further contribute to fibrosis generation in the liver as a previous study suggested (Chiang et al., 2011). Therefore, we investigated genes involved in fibrosis. Among genes having an impact on fibrosis, we observed significantly elevated methylation levels in the promoter regions of *Collagen 1A1* (*COL1A1*) (Figure 4A, FC = 1.33 in FFC vs. SD diet and FC = 1.32 in FFC vs. FFC/SD diet) and β*-Klotho* (*KLB*) (Figure 4B, FC = 2.00 in FFC vs. SD diet and FC = 1.74 in FFC vs. FFC/SD diet) in the FFC group. However, hypermethylation of promoter regions of these two genes exhibited differential functions on transcriptome regulation. qPCR analysis revealed that the expression level of *COL1A1* was significantly higher in the FFC group (FC = 2.68 in FFC vs. SD diet and FC = 1.92 in FFC vs. FFC/SD diet; *P* < 0.05) (Table 3), whereas the expression level of *KLB* was repressed, as significantly decreased expression level in the FFC group was observed when comparing with the SD and the FFC/SD pigs (FC = −20.68 in FFC vs. SD diet and FC = −13.87 in FFC vs. FFC/SD diet; *P* < 1.0E-5) (Table 3). These results suggest antagonistic activity of these two genes during fibrosis generation.

### Genes Still Perturbed After Diet Reversion

**Figure 5:**
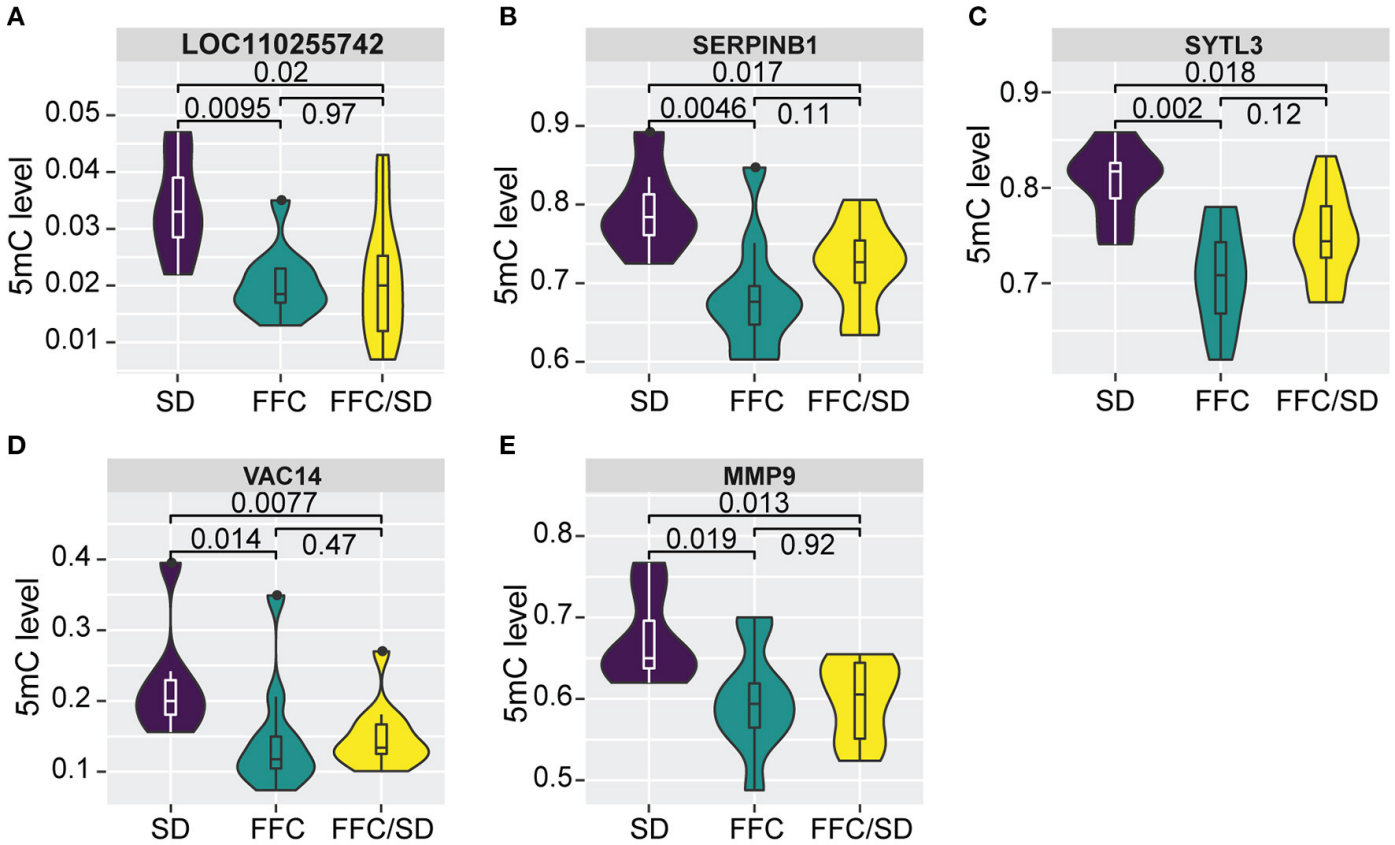
Differential methylation profiles after diet reversion. **(A–E)** Boxplot of methylation level of promoter regions of *LOC110255742* **(A)**, *SERPINB1* **(B)**, *SYTL3* **(C)**, *VAC14* **(D)**, *MMP9* **(E)**. *P*-values were calculated using Mann-Whitney *U*-test.

The five genes identified with differential methylation even after diet reversion exhibited hypomethylation in FFC and FFC/SD diet fed pigs in comparison to SD pigs (Figures 5A–E). However, only *MMP9* was investigated by qPCR. As expected, the expression of *MMP9* in FFC/SD diet group was upregulated 3.7-fold, while in FFC diet group *MMP9* was 6.1-fold up-regulated when comparing to SD group (*P* < 0.01) (Table 3).

## Discussion

Establishing and maintaining proper DNA methylation patterns provides a link between environmental factors and gene expression, and thus play a pivotal role for health (Ge et al., 2014). In this study, we have profiled the dynamic responses of promoter methylomes following high fat/fructose/cholesterol diet-induced changes in liver to characterize a Göttingen Minipig model of obesity, and further evaluated its reversibility after changing the diet to normal chow. Our results support the notion that DNA methylation may play a role in environmentally-induced transcriptional response impacting pathological processes in obesity.

As shown in Table 1, the FFC pigs developed not only increased levels of body weight and total body fat, but TG and TC levels in plasma were also elevated, which are characteristic features of dyslipidemia, suggesting that pigs in the FFC group became morbidly obese (Schumacher-Petersen et al., 2019). Interestingly, these morphometric and metabolic features induced by the FFC diet was only partially reversed by changing the diet to normal chow. This can suggest that diet components are the main factors for triggering obesity onset, but once the disease has progressed, it is difficult to restore morphometric and metabolic features back to normal levels by reversing the diet. Our results are in support of previous findings in juvenile Ossabaw swine showing that Western-style diets could induce pathological changes, including obesity, dyslipidemia, and systemic insulin resistance (Panasevich et al., 2018). As the diet for the Göttingen Minipigs had a reduced content of cholesterol (from 2 to 1%) in the last period of feeding (9G4U), the histological features in the two studies appear to some differences. There might be more steatosis in the Ossabaw pigs, but they have not shown any ballooning, suggesting that individual roles of dietary components may be of differential significance on the development of obesity and NASH. This was evidenced by association between dietary fructose and pathophysiology of chronic diseases, e.g., NAFLD, hypertension, and cardiovascular disease, which have recently attracted considerable interest due to fructose metabolism occurring mainly in liver (Akram and Hamid, 2013). These results suggest that diet components are the main factors for triggering obesity onset and progression.

As the DNA methylation process is mainly dependent on dietary methionine supply, we speculate that dietary changes might influence distribution of the DNA methylomes of different feeding groups. In our study, we found that there was no difference on the mean of promoter methylomes among the three diet groups. This was due to the fact that the absolute values of the FCs of the hypomethylated DMRs were higher than the FCs of the hypermethylated DMRs (Supplementary Figure 5), even though ~2-times as many hypermethylated DMRs were identified. As DNA methylation scenarios are heterogeneous between cells, which may result from heterogeneity within a sample, the measured methylation represents a mixture of unmethylated and methylated alleles (Mikeska et al., 2010), thus resulting in small changes of those differential methylated promoters (1.06 to 2.60-fold). When looking at the effect of the FFC diet compared to the SD and FFC/SD diets, we found methylation changes in functionally relevant genes for obesity pathogenesis, e.g., the three hypermethylated genes involved in the lipid/cholesterol metabolic pathways, including *HMGCR, LDLR*, and *MTTP*. Indeed, deregulation of hepatic cholesterol metabolism plays an important role in the development and progression of obesity, especially for *HMGCR* and *LDLR*, which are the pivotal rate-limiting enzymes controlling cholesterol synthesis and homeostasis (Nestel et al., 1973). Different from *LDLR*, promoters of *HMGCR* exhibited low DNA methylation levels in the SD group, which may be of clinical importance as previous study suggested (Mikeska et al., 2010). We observed that the FFC diet induced 1.72-fold and 2.25-fold methylation in *HMGCR* and *LDLR*, respectively. In agreement with our results, Cai and coworkers found 2.2-fold higher methylation levels in the promoters of *HMGCR* and *LDLR* in liver of piglets born from betaine-supplemented sows compared to normal piglets (Cai et al., 2014). In accordance with promoter hypermethylation being associated with gene repression, our results show significantly decreased expression of these two genes in FFC pigs. Also, the *MTTP* promoter exhibited 1.18-fold higher methylation under FFC diet intervention. Considering the functional roles of *MTTP* in very-low-density lipoprotein (VLDL) assembly/secretion in hepatocytes, the diet-induced decreased expression of *MTTP* might promote liver steatosis through elevated formation of VLDL particles (Wang et al., 2014). Similar gene regulation results were also obtained from porcine lymphocytes in obesity, which showed perturbation of lipid metabolism and inflammatory response (Jacobsen et al., 2016).

Obesity is characterized by a chronic low-grade inflammation, which results in increased levels of inflammatory mediators, such as cytokines and adipokines. These mediators regulate various functions including metabolic energy balance, inflammation, and immune response (Asrih and Jornayvaz, 2013). Of the two genes investigated by qPCR (*TNF* and *TLR4*), methylation changes of *TLR4* (FC = −1.15 in FFC vs. SD diet and FC = −1.33 in FFC vs. FFC/SD diet) may be of functional significance as reflected by the significantly higher expression of *TLR4* in the FFC group when comparing with the FFC/SD and SD groups. The role of *TLR4* in liver disease is well established as an activator of obesity-associated inflammation and insulin resistance, thus serving as a therapeutic target for the treatment of type 2 diabetes (Jia et al., 2014). Obesity triggers low-grade chronic inflammation which can act as risk factor for obesity-related complications, such as NAFLD (Greenberg and Obin, 2006). However, as described in Schumacher-Petersen et al. (2019) and Cirera et al. (2020), in spite of the metabolic challenges imposed by the FFC diet, hepatic steatosis and other key human pathological findings characterizing NAFLD are lacking in this GM model.

The increased inflammation and impaired lipid and cholesterol metabolism resulting from methylation changes observed in this study may have impact on generation of fibrosis in the liver (Chiang et al., 2011; Ahmed et al., 2012). With respect to this, we observed *COL1A1* and *KLB* both exhibiting significantly higher methylation levels in the FFC group compared to the SD and FFC/SD groups. Epigenetic evidence of liver *COL1A1* induced by a high-fat diet has been investigated in SM/J mice, which exhibited differential methylation spanning exons 23 and 24 of the gene (Keleher et al., 2018). Methylation levels of *KLB* have been studied in pure adipocyte fractions from obese pigs, showing strong methylation-induced expression silencing in intron 4 (Jacobsen et al., 2019). Moreover, the expression levels of these two transcripts (*COL1A1* and *KLB*) showed opposite trends in FFC compared to the other two groups. *COL1A1* is a pro-fibrogenic gene proposed as a reliable biomarker and putative therapeutic target for hepatocellular carcinogenesis (Ma et al., 2019). The upregulation of *COL1A1* induced by dietary challenge suggests its potential involvement in liver fibrosis. Anti-fibrotic effects of β-klotho have been proved in several tissues and klotho-deficient mice spontaneously develop fibrosis and exacerbate the disease progression (Mencke et al., 2017). Further, patients with perturbed inflammatory response and fibrogenesis also exhibited decreased expression level of β-Klotho via the NF-κB and JNK pathway (Lee et al., 2018). These data indicate antagonistic activity of these two genes during fibrosis generation.

Obesity affects metabolism and it has been shown that obesity-associated methylation was only partially reversible and restricted to a subset of loci (Kim et al., 2019). In human studies, an isocaloric low-carbohydrate diet is a widely recommended intervention for treating obesity and NAFLD (Mardinoglu et al., 2018). This dietary regime can result in marked reductions in body weight and further promote microbial shifts toward folate-mediated one-carbon metabolism. Therefore, therapeutic interventions leading to massive weight loss, e.g., bariatric surgery, can effectively reverse the pathological states of NAFLD and NASH patients (Laursen et al., 2019). In the present study, we found substantial weight loss in the FFC/SD pigs, indicating that the liver pathology improved after diet reversion. We observed that the perturbation of the methylation pattern of the majority of DMGs (*N* = 125) could be reversed to normal after diet reversion, and this reversibility was accompanied by normalization of expression of several key metabolically-related genes, e.g., *HMGCR, LDLR*, and *KLB*, etc. However, the fibrosis-related gene *MMP9* still maintained differential methylation in the promoter region when comparing the FFC/SD vs. SD groups even after 6 months of diet normalization. *MMP9* encodes a zinc-dependent endopeptidase implicated in degrading extracellular matrix, immunity, and especially contributes to pathogenetic mechanisms involved in the development of obesity (Ludvigsen et al., 2019). Furthermore, *MMP9* is tightly associated with liver fibrosis as it can cleave different types of collagen besides other basal membrane proteins (Zbodakova et al., 2017). This perturbation of *MMP9* in FFC and FFC/SD pigs is in agreement with our morphometric and metabolic findings that FFC diet-fed pigs developed characteristic features of fibrosis, inflammation and cytoplasmic alterations in the liver, whereas features of fibrosis in the FFC/SD group persisted when comparing to the SD group (Schumacher-Petersen et al., 2019).

In conclusion, we show that high fat diet intervention predisposed the liver to pathological processes and triggered differential methylation and expression of key genes of relevance for metabolism in the liver. Of note, the majority of the perturbed DNA methylation patterns could be reversed by diet reversion. Our findings provided here have clinical implications for identifying potential epigenetic biomarkers for treatment of obesity in humans.

## Data Availability Statement

The datasets presented in this study can be found in online repositories. Raw LHC-BS sequencing data were deposited in the GEO with the following accession number: GSE155197.

## Ethics Statement

All experimental protocol involving animals have been reviewed and approved by the Danish National Committee on Animal Experimentation.

## Author Contributions

FG and MF conceived the project. YF and FG analyzed and interpreted the LHC-BS data. YL performed the LHC-BS library construction. SC and ET performed the qPCR validation. LO, BC, HP, TL, RK, and CS-P designed and carried out the animal experiment. RK and CS-P did the biochemical and histological analyses of the liver tissue. YF was the major contributor in writing the manuscript. FG, MF, SC, and YD critically reviewed the manuscript. All authors read and approved the final manuscript.

## Conflict of Interest

The authors declare that the research was conducted in the absence of any commercial or financial relationships that could be construed as a potential conflict of interest.
