# Supplementary material for: Diet-Dependent Changes of the DNA Methylome Using a Göttingen Minipig Model for Obesity
Source: Front Genet. 2021 Mar 11;12:632859. doi: 10.3389/fgene.2021.632859 (PMC7991730; doi:10.3389/fgene.2021.632859)
Supplement: Supplementary file 5 [file Data_Sheet_1.docx]

**Diet-dependent changes of the DNA methylome using a Göttingen Minipig model for obesity**

**Supplementary Table legends:**

**Supplementary Table S1.** Data generation for LHC-BS.

**Supplementary Table S2.** Methylation level of promoter regions of the 160 genes that showed differential methylation both in SD and FFC/SD groups when comparing with FFC group.

**Supplementary Table S3.** Pairwise Pearson’s correlations between methylation levels of promoter regions of core genes (*N* = 160) and morphometric parameters.

**Supplementary Table S4.** Potential gene ontology (GO) and enrichment analysis of differentially methylated genes.


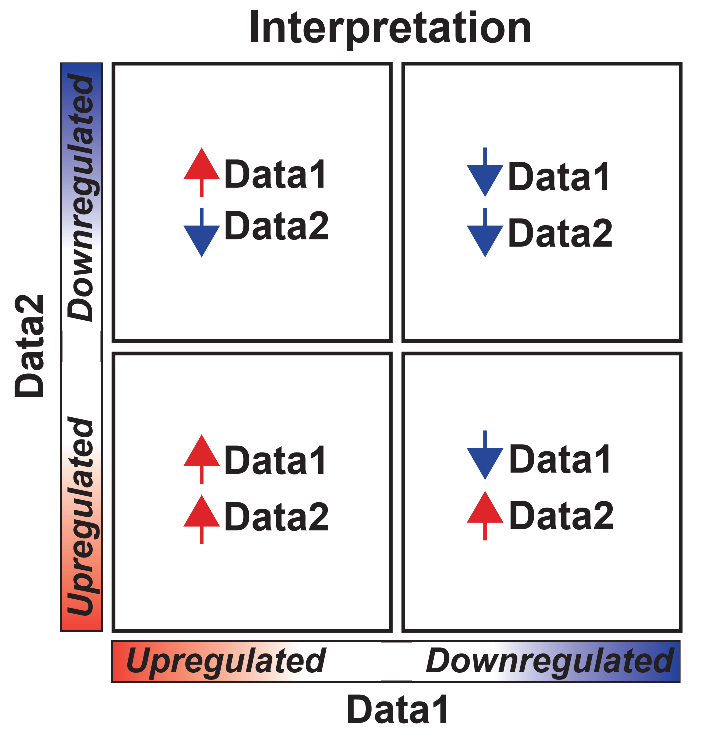


**Supplementary Fig. 1** Schematic indicating interpretation of Rank-Rank hypergeometric Overlap test plots. For example, a hot spot in the bottom left corner represents overlap in genes upregulated both in data1 and data2; and a hot spot in the bottom right corner represents overlap in genes upregulated in data1 and downregulated in data2.


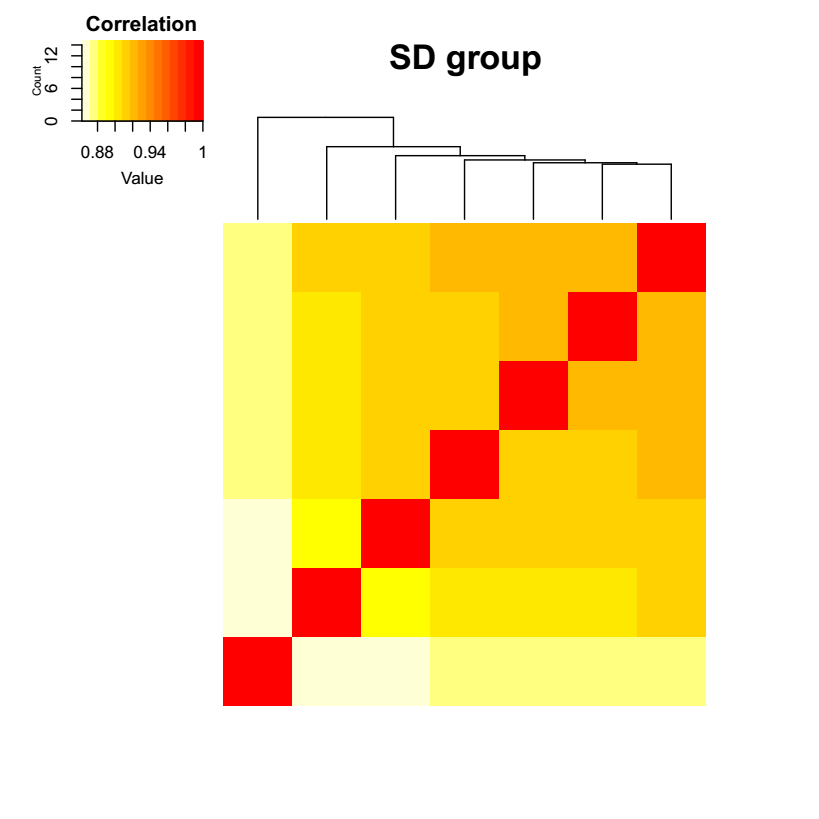

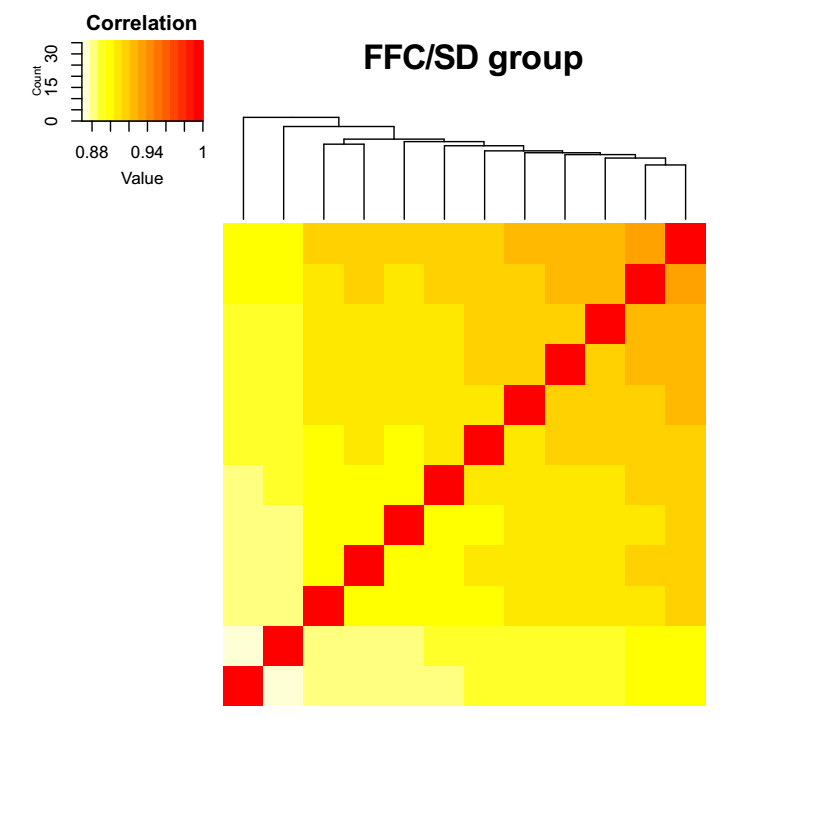

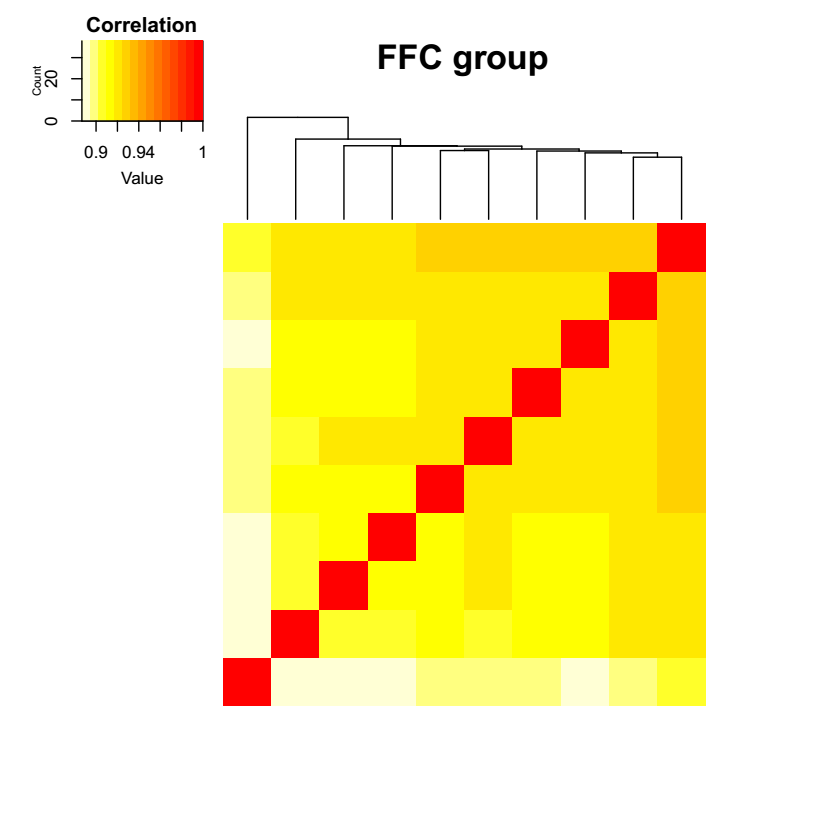


**Supplementary Fig. 2** Heatmap of pairwise Pearson’s correlation coefficients between individuals based on the 408,458 methylated CpG sites in all samples of each diet feeding group.


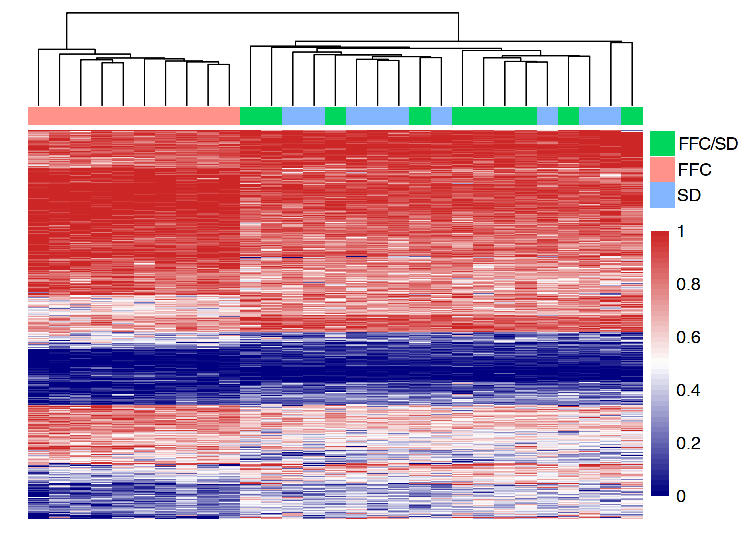


**Supplementary Fig. 3** Hierarchical clustering analysis based on the top 1000 genes containing highly variable promoter methylations based on the *P*-values obtained from chi-square analysis. The chi-square analysis was performed using ‘chisq.test’ package in R (version 3.6.2) based on the mean methylation level of common promoter regions of three diet groups.


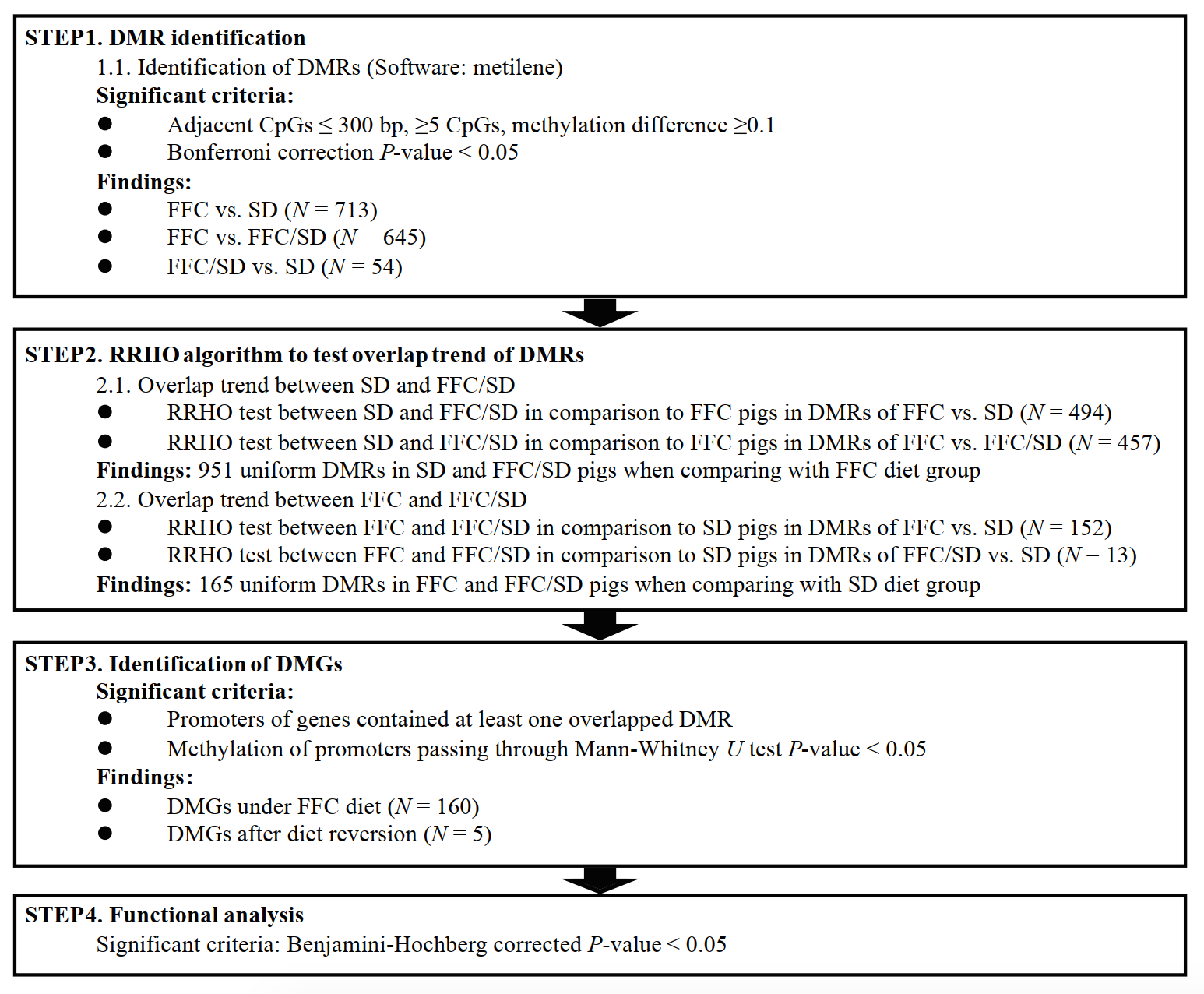


**Supplementary Fig. 4** Flowchart of DMG analysis process.


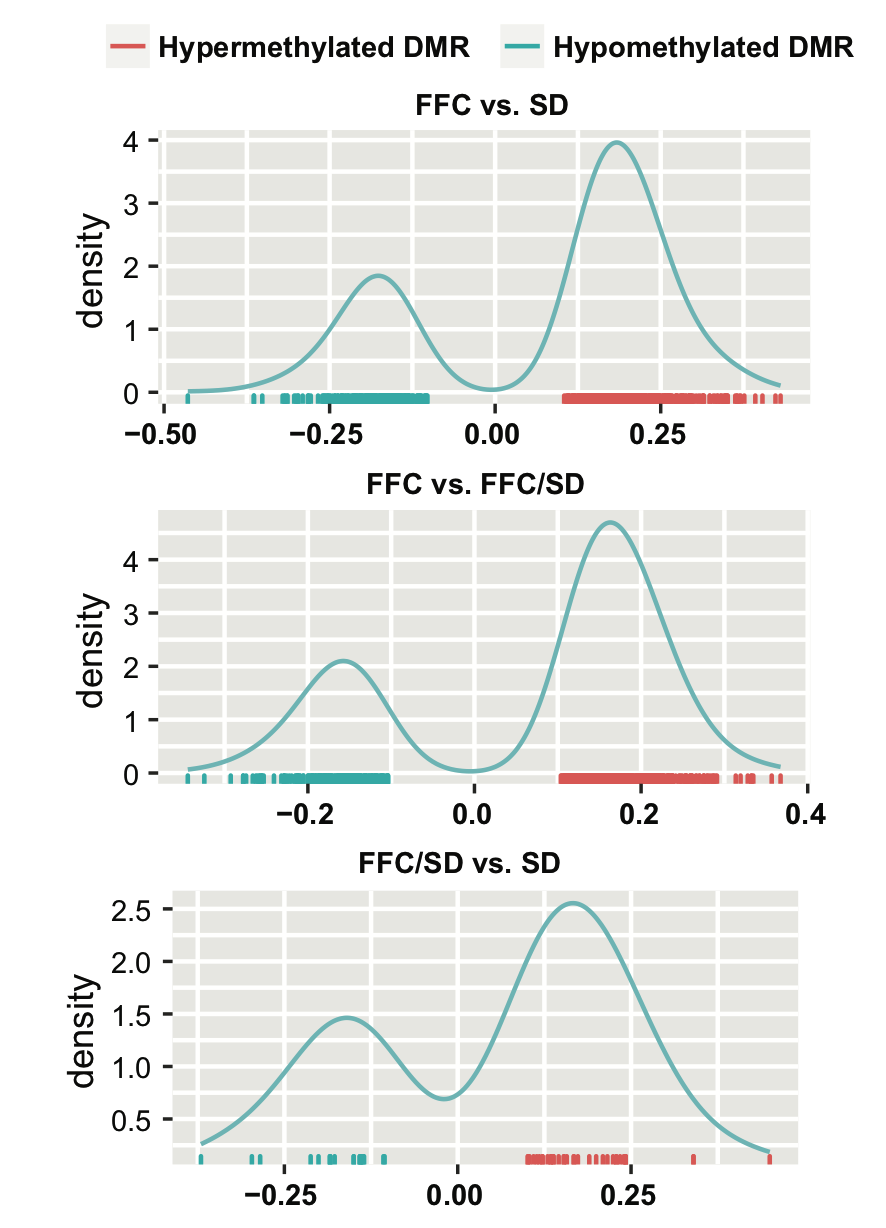


**Supplementary Fig. 5** Density of methylation differences between hypermethylated and hypomethylated DMRs in corresponding DMR regions of FFC vs. SD, FFC vs. FFC/SD and FFC/SD vs. SD.
